# The function and regulation of ADP-ribosylation in the DNA damage response

**DOI:** 10.1042/BST20220749

**Published:** 2023-05-12

**Authors:** Lena Duma, Ivan Ahel

**Affiliations:** Sir William Dunn School of Pathology, University of Oxford, Oxford, U.K.

**Keywords:** ADP-ribosylation, DNA synthesis and repair, PARP

## Abstract

ADP-ribosylation is a post-translational modification involved in DNA damage response (DDR). In higher organisms it is synthesised by PARP 1–3, DNA strand break sensors. Recent advances have identified serine residues as the most common targets for ADP-ribosylation during DDR. To ADP-ribosylate serine, PARPs require an accessory factor, HPF1 which completes the catalytic domain. Through ADP-ribosylation, PARPs recruit a variety of factors to the break site and control their activities. However, the timely removal of ADP-ribosylation is also key for genome stability and is mostly performed by two hydrolases: PARG and ARH3. Here, we describe the key writers, readers and erasers of ADP-ribosylation and their contribution to the mounting of the DDR. We also discuss the use of PARP inhibitors in cancer therapy and the ways to tackle PARPi treatment resistance.

## Introduction

DNA plays an essential role in cell information storage. It contains the blueprints for the synthesis of the molecules necessary for life. Therefore, maintenance of this molecule is essential for organisms’ survival. DNA's unique role means that it cannot be replaced when damaged, as other macromolecules are. Instead, it needs to be repaired. To this end, multiple mechanisms have evolved to repair the different types of damage that DNA can sustain. As with most cellular functions, DNA damage repair is regulated through post-translational modifications (PTMs), which allow for tight temporal control of the response. Many different PTMs are involved in the DNA damage response (DDR) and understanding their dynamics helps our understanding of how the cell maintains genomic stability. Failure to successfully maintain the genome integrity can lead to the development of various disease states. Deficiencies in DDR have been indicated to be involved in carcinogenesis, immunodeficiency and neurodegeneration [1,2]. Thus, understanding the players involved in mounting a successful DDR is recognised to be of great importance in the biomedical sciences community.

A PTM important in the DDR is ADP-ribosylation, a reversible covalent attachment of ADP-ribose (ADPr) moieties to proteins. In humans, the largest protein family that can synthesise ADPr are Poly(ADP-ribose) Polymerases (PARPs) [3]. ADPr can be added in chains (PAR) or as a single molecule (MAR). The PAR chains are more transient and the hydrolytic activity of the Poly(ADP-ribose) Glycohydrolase (PARG) enzyme swiftly turns them back into mono-ADPr tags that can stay on chromatin for longer [4,5]. The ADPr modification can be placed on different amino acids on the protein. While serine is the most common target in DDR [6–10], glutamate/aspartate is also modified [11,12] and some studies suggest tyrosines as possible sites [13–15]. The PAR chains can be branched and there is some evidence that the length and branching of the chain have implications for the functionality of the cell, suggesting the existence of a ‘PAR code’ [16–18]. ADPr modification could be also remodelled by the addition of an ubiquitin moiety which is catalysed by the deltex family of E3 ligases [19]. Altogether, ADPr is a very diverse modification that regulates many different processes including transcription, metabolism, immune response and DDR [20–23]. ADPr has been suggested to play a role in almost all DDR pathways. In fact, it has been demonstrated that upon DNA damage, one-third of the proteins present in the nucleus are ADP-ribosylated, mostly on serine residues [24]. All of this taken together makes understanding ADPr signalling critical in understanding way in which cells maintain genome integrity.

## DNA damage response PARPs

**Figure 1.**
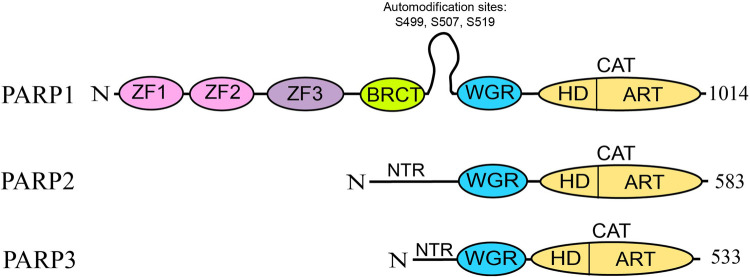
Domains of PARP1-3. The number of amino acids indicated on the right. ZF, Zinc-finger; BRCT, BRCA1 C-terminal domain; WGR, nucleic acid binding domain; HD, helical domain; ART, ADP-transferase domain. Adapted from Luscher et al. [3].

Of all the 17 PARP family proteins, only PARP1–3 act as DNA damage sensors. PARP1 and PARP2 can catalyse the formation of PAR chains on proteins and have a partially redundant function [25,26]. PARP1 is thought to be the most robust ADPr writer in response to DNA damage. It is a very abundant nuclear protein and is responsible for the majority of ADPr upon exposure to DNA-damaging agents [27,28]. PARP1 is the founding member of the family as it was the first one to be successfully purified and characterised [29–31]. The protein contains a variety of domains: three zinc finger domains on the N-terminal which allow for binding to DNA breaks; a WGR domain which also interacts with the DNA; a BRCT domain which can interact with DNA, PAR or other proteins; an automodification loop and a catalytic domain on the C-terminal (Figure 1) [32–35]. The catalytic domain contains an autoinhibitory domain which prohibits PARP1 enzymatic activity unless it is bound to DNA breaks [36–39]. PARP1 is a DNA damage sensor which is quickly recruited to the site of damage and PARylates histones, itself and other surrounding proteins to create a scaffold to recruit DDR factors. PARP1 recognises the breaks in the DNA backbone and binds to them [40]. Since the DNA backbone is disrupted in a variety of situations, PARP1 can be involved in different DDR pathways [41].

PARP1–3 are activated by both single- and double-stranded DNA breaks [38,42–44]. The breaks may arise directly, as intermediates in various DDR pathways such as Base Excision Repair (BER) or as intermediates in replication. In all cases, PARP1 binds to the DNA which triggers a conformational change in its autoinhibitory HD domain [32,45]. This change uncovers the NAD^+^ binding site, allowing for efficient NAD^+^ binding and hydrolysis [46]. After binding to the DNA, PARPs can effectively PARylate themselves and neighbouring histones to recruit DDR factors to the site. The PAR chains can be recognised by a variety of reader domains. One of the key readers of PAR in the context of DDR is X-ray repair cross-complementing protein 1 (XRCC1), a scaffold protein which recruits DNA repair ligase and DNA end processing factors to DNA breaks [47]. Another aspect of PARP-regulated activity during DDR is regulation of chromatin structure. Upon DNA damage, chromatin relaxes in a PARP1-dependent manner. This is thought to occur through the activity of ALC1, a helicase, and Aprataxin-and-PNK-like factor (APLF), a histone chaperone, amongst other actors [48–52].

However, loss of PARP1 does not result in a dramatic phenotype of genomic instability [53]. This is likely due to the presence of PARP2 activity in the cells. Double knockout of PARP1 and PARP2 leads to embryonic lethality in mice, demonstrating that the enzymes have somewhat redundant activities [54]. PARP2 is a PARP1 homologue that lacks the N-terminal Zinc finger domains of PARP1 (Figure 1). Instead, it contains an unstructured N-terminal region (NTR) whose function in DDR is not fully defined, though it has been suggested that NTR plays a role in PARP2 recruitment to PAR [55,56]. However, PARP2 is still able to bind to DNA breaks through its WGR domain [44,55]. The dynamics of PARP2 association with DNA breaks are different from those of PARP1. PARP2 recruitment to the breaks is slower and it is retained on the damage site longer than PARP1 [57]. These subtle differences suggest that, while PARP1 and PARP2 roles in signalling overlap they contribute differently to the synthesis of PAR chains. PAR synthesised by PARP1 has been demonstrated to activate PARP2 which then synthesises branched polymers on the linear PARP1-derived PAR chains [56]. Currently, very little is known about how the branching is controlled and how it contributes to the recruitment of different DNA damage factors [17]. Additionally, cryo-EM structural studies suggest that PARP2, together with its accessory protein HPF1, is able to bridge the double-stranded break and hold the two strands together acting not only as the sensor of the break but also contributing to the first stage of the repair process [58]. This might explain why PARP2 is reported to stay longer on the chromatin after DNA damage.

Similarly to PARP2, PARP3 contains only the NTR, WGR and the catalytic domain (Figure 1) but unlike PARP2, it is only capable of attaching a single ADP-ribose moiety to proteins [44,59]. The role it plays in DDR is not clear but it has been reported to act in both single-stranded break (SSB) repair and Non-Homologous End Joining (NHEJ) [60,61]. A potential function in gene regulation has also been proposed [62]. In DDR, it is known to MARylate some of the key proteins such as Ku70 and PARP1, as well as histones [59,60,63]. Its interactions with Ku-proteins and PARP1 seem to be important for the regulation of the c-NHEJ pathway for repairing DSBs [61,64]. The exact mechanisms by which PARP3 participates in those pathways remain to be elucidated.

## Serine is the most common ADP-ribosylation site

Historically, PARP1-3 were thought to primarily modify aspartate and glutamate residues, though Glu/Asp-linked ADPr has been mechanistically not well understood [12,26,65,66]. In the last few years, it has been demonstrated that ADP-ribosylation of serine residues in proteins is the most common form of ADPr upon DNA damage [6] and it predominantly targets proteins relevant for genome stability. It was likely unnoticed throughout the years because *in vitro* PARP1/2 almost exclusively PARylate glutamate and aspartate, and therefore serine-ADPr was not considered a possibility during earlier mass spectrometry studies. This changed with the discovery of Ser-ADPr in cells and the characterisation of the Histone Parylation Factor 1 (HPF1), an accessory protein which interacts with PARP1/2 [67,68]. The HPF1 makes a composite active site together with PARP1 or PARP2 which dramatically increases the efficiency of DNA damage induced ADP-ribosylation and changes PARP specificity to allow for PARylation of serine residues [8,67,69]. This change of specificity is possible because HPF1 completes the catalytic domain of PARP1 providing an additional glutamate residue which allows for deprotonation of serine. This is necessary for serine modification but does not happen during aspartate/glutamate ADP-ribosylation [69,70]. Through this, HPF1 interacts with PARP1/2 to promote PARylation of histones and facilitate DDR [8,9]. In line with its function, loss of HPF1 is associated with sensitivity to DNA-damaging agents such as methyl methanesulfonate (MMS) as well as PARP inhibitors [35,67]. As well as switching PARP 1/2 specificity towards serine, HPF1 limits the enzyme's ability to form PAR chains through steric hindrance of the binding pocket necessary for the recognition of the new acceptor NAD^+^ during chain elongation [67,69]. This results in two distinct steps in the PARylation process in DDR: HPF1-dependent initiation of the chain; and HPF1-independent elongation [71]. This model is supported by data showing that once histones have been primed by HPF1/PARP1 complex, PARP1 is sufficient to create PAR chains [71,72]. New data suggests that by promoting shorter chain synthesis on the histones, HPF1 promotes PARP1-dependent chromatin relaxation during DDR [73]. Finally, HPF1 restricts PARP1 NAD glycohydrolase activity [74].

In addition to PARylating histones and other proteins in trans, PARP 1/2 also heavily automodify themselves upon DNA damage [8,75]. This activity can act partly as further scaffolding for the recruitment of DDR factors but it is mainly a part of negative feedback loop. AutoPARylation is key for PARP1/2 dissociation from the DNA which allows the repair factors to reach the break site. There are three serine residues on PARP1 that are key targets of automodification [8,35]. This mechanism is exploited by PARP inhibitors which, by blocking PARP 1/2 activity, take away its ability to dissociate from the break site — effectively ‘trapping’ it on the DNA [35,76–78]. This prevents the repair factors from successfully repairing DNA and leads to further breaks upon the collapse of the replication and transcription machinery with the trapped PARPs [77–80].

PARP/HPF1 also targets for Ser-ADPr many other proteins involved in genome stability, including various DNA repair factors, and proteins involved in chromatin structure regulation, transcription, RNA metabolism and mitosis [9,81]. It has been shown that HPF1-dependent ADPr is necessary for the recruitment of DNA Ligase III to Okazaki fragments [82]. And since many other proteins have been reported to be ADP-ribosylated on serine residues [8,9,14], it is likely that HPF1 and Ser-ADPr play an important role in different repair pathways and various aspects of genome stability. It is unclear how the PARP1/2 interaction with HPF1 is regulated. One proposed mechanism is through HPF1-dependent ADP-ribosylation by PARP1 [15]. Also, other factors could modulate PARP1/HPF1 interaction or activity such as the mitotic factor TPX2 and CARM1 [83,84].

Additionally, there is an emerging new target for ADPr — nucleic acids [85,86]. *In vitro* studies have shown that mammalian DDR PARPs are capable of modifying DNA breaks at phosphate groups [87–91]. Additionally, low levels of adenosine PARylation have been reported in human cells. This modification is likely catalysed on the bases in single-stranded DNA rather at the terminal phosphates as reported previously [92]. It has also been demonstrated that TARG1, a mammalian ADPr hydrolase, is capable of cleaving DNA–ADPr in cells [93]. It is currently unclear whether this activity of PARPs has any physiological roles or if it is an off-target, pathological event. However, the fact that a variety of mammalian hydrolases have been observed to be capable of removing ADPr from DNA [87] and that reversible DNA–ADPr signalling has been characterised in bacteria [94] suggests a potential for DNA–ADPr to play a role in DDR signalling. Suggested roles for this process include the protection of the DNA ends from nuclease activity or the inhibition DNA repair during the apoptotic response [87]. Further studies are necessary to determine whether DNA–ADPr is a lesion or a normal part of the DDR.

## Readers of ADPr

As explained above, PARP1 acts as a sensor of DNA damage and it ADP-ribosylates histones to recruit DDR factors. Apart from better characterised ADPr/PAR-binding domains; PBM, PBZ, WWE and macrodomain [95], domains such as Forkhead-associated (FHA) or BRCA1 C-terminal (BRCT) can also interact with ADPr [96,97]. Many of these domains were originally discovered as readers of protein phosphorylation or nucleic acids and have also now been confirmed to interact with PAR. This variety of ADPr-recognizing domains allows for the recruitment of numerous factors important for DDR.

XRCC1 is a scaffold for DNA repair ligase III complex and is involved in single-strand break repair, BER and Nucleotide Excision Repair (NER) [98]. It interacts with DNA ligase via its C-terminal BRCT domain [99,100] and with DNA polymerase beta via its N-terminus [101]. It has a phosphorylated epitope that binds to either Aprataxin, APLF or polynucleotide phosphatase/kinase (PNKP) [98]. XRCC1 is capable of binding ADPr through its first BRCT domain [102,103]. Consequently, XRCC1 is quickly recruited to the DNA damage site in a PARP1/2-dependent manner [104].

APLF is an ADPr reader with a PBZ domain on the C-terminus [105]. It accumulates on the DSB sites in a PARP1-dependent manner [106]. While some studies report that it primarily binds PARP2-dependent branched ADPr, others have observed preferential binding to linear chains [17,56]. It is suggested to work with PARP3 to resolve γH2AX foci and thus aid in the repair of DSBs [61]. APLF can interact with both XRCC1 and XRCC4 [61,107]. It promotes the retention of the XRCC4/DNA ligase IV complex in chromatin thereby promoting repair by non-homologous end joining proteins [61]. In addition to being involved in the recruitment of DDR factors, APLF has been observed to act as a histone chaperone [50,108,109], although the exact role of this activity in DDR is not fully understood.

ALC1 (also referred to as Chromodomain-helicase-DNA-binding-protein-1 like (CHD1L) in the literature) is a chromatin remodelling enzyme that requires adenosine triphosphate (ATP) to induce chromatin relaxation in response to DNA damage [48]. It contains a macrodomain and has been demonstrated to be recruited by PARP1/2 in response to laser irradiation and UV radiation *in vivo* [48,110,111]. ALC1 binds to PARylated nucleosomes and its chromatin remodelling activity is crucial for DDR: the relaxation of chromatin is key to allowing DDR factors to gain access to the DNA damage site [49,112,113]. ALC1 loss is synthetically lethal with the defects in repair by HR, as ALC1 loss is associated with chromosome instability caused by unrepaired DNA gaps at replication forks [114]. ALC1 overexpression leads to sensitivity to X-irradiation and has been associated with cancer progression [115]. ADP-ribosylation can be recognised by a variety of other DDR factors and detailing all of them is beyond the scope of this review [96,116].

## Erasers of ADPr

**Figure 2.**
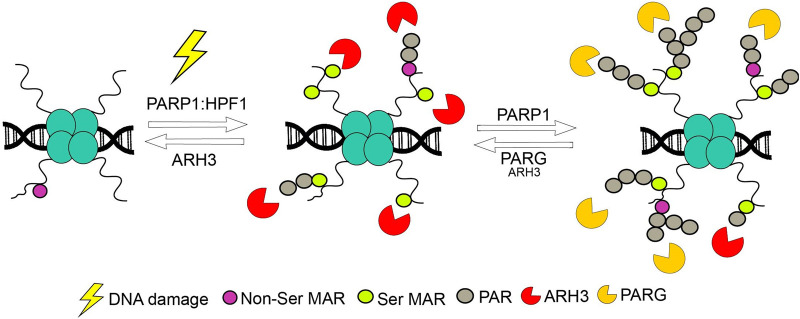
ADPr homeostasis. Synthesis of ADPr during DDR occurs in two steps. First, HPF1/PARP1 complex MARylates serines. Then, PARP1 on its own creates long chains that recruit DDR factors. Degradation of PAR follows a similar pattern, long chains are degraded primarily by PARG with assistance of ARH3. However, as the chains become shorter ARH3 takes over. Only ARH3 is capable of removing MAR from serine. Adapted from Prokhorova et al. [71].

ADPr in response to DNA damage is under tight temporal control. The PAR chains only stay on the chromatin long enough to recruit the downstream factors to the site. Afterwards, they need to be removed from the area so that the factors can interact with the damaged DNA and repair it [117]. This fine control requires specific factors that can remove the ADPr signal in a timely manner. This is also important because long PAR chains are toxic to the cell long term, as it progresses through the cell cycle [71]. The production of ADPr is also metabolically costly because of the consumption of NAD^+^ associated with the process. The energetic cost can be compensated by the quick degradation of the chains which releases free ADP-ribose that can be converted into ATP [118–120]. A variety of ADPr hydrolases present in the cells for that purpose. The hydrolases can be divided into two groups: macrodomain hydrolases and ARH-type hydrolases [121]. PARG contains a macrodomain which can recognise the ribose-ribose linkage between two ADP-ribose units and cleave the bond [122–124]. This way, it can degrade the PAR chains with high efficiency. However, it is not able to remove the final ADPr that anchors the chain to the target protein [117,122,125]. This step can be performed by (ADP-ribosyl)hydrolase 3 (ARH3) [4]. That enzyme is evolutionary unrelated to PARG [126,127] and cleaves PAR chains with lower efficiency than PARG [128] but can also remove the final ADPr from the protein [4]. ARH3 is especially relevant for DDR as it is the only ADPr hydrolase that can ADPr from serine residues, where the majority of ADPr is placed upon DNA damage. Through these differing activities, the two enzymes work together to remove the ADPr chains from proteins. PARG degrades the majority of the poly-ADPr but its activity becomes lower as the chains become shorter [125]. ARH3 becomes more prominent on short chains and it removes the final unit from the protein [71,125]. This two-step degradation process of Ser-linked PAR fits together with the two-step synthesis process that was detailed earlier (Figure 2) [71]. Other hydrolyses are involved in removal of ADPr from other amino acids, for example, glutamate ADPr is reversed by terminal ADP-ribose glycohydrolase (TARG1), MACROD1 and MACROD2 [129–131].

PARG activity is essential for development in both mice and flies [132,133]. *ARH3* is not an essential gene but loss of ARH3 activity is associated with stress-induced childhood-onset neurodegeneration with variable ataxia and seizures (CONDSIAS) disorder [134–138]. The neurodegeneration is usually triggered by periods of stress or an infection. This suggests that PAR-degrading potential of ARH3 becomes particularly needed upon additional stress which triggers increased PARP activity and therefore increased PAR levels [136,137]. ARH3 deficiency in cells leads to oxidative stress sensitivity and telomere defects [71,139]. Mechanistically, it was proposed that ARH3 protects the cells from parthanatos — a PAR-associated programmed cell death. This is characterised by the overactivation of PARP1/2 upon significant DNA damage leading to the synthesis of long chains of PAR. These chains are then cleaved by PARG and released into the cytoplasm where they act as a death signal leading to the release of apoptosis-inducing factor (AIF) from the mitochondria [138,140]. ARH3 present in the cytoplasm is thought to counteract this by degrading the free-PAR that was released from the nucleus [139]. Without ARH3 present in the cell, lower levels of free-PAR are necessary to trigger parthanatos which would explain the CONDSAIS phenotype [141]. ARH3 is also located in mitochondria but the neurodegeneration phenotype appears to be due to its nuclear/cytoplasmic function [134,142]. Loss of another ADPr hydrolase, TARG1, has also been associated with neurodegeneration, highlighting the importance of proper ADPr homeostasis for cell viability [129]. TARG1 is a macrodomain type hydrolase related to PARG which is capable of cleaving ADPr linked to glutamate [129]. Through this activity it can also release long PAR chains into the cytoplasm, possibly contributing to parthanatos.

## Targeting PARP signalling in cancer therapy

Since PARP activity is so important for initiating a successful DDR, inhibitors of PARPs have been developed to selectively target Breast Cancer gene (BRCA)-deficient tumours [143–148]. This is possible because the inhibition of PARP activity decreases PARP's ability to dissociate from the DNA, resulting in DNA–PARP complexes which induce DSBs upon replication [76,149]. BRCA-deficient cells do not possess efficient Homologous Recombination (HR) that would be able to repair the breaks which explains the selectivity of the drug [78]. The trapping occurs because autoPARylation is necessary for PARP dissociation from the DNA, hence the inhibition of PARP causes the persistence of PARP on the DNA [35,76]. These observations have led to the development of numerous PARP inhibitors (PARPi) which inhibit PARP activity by competitive binding to the NAD^+^ binding pocket [78,147,150,151]. However, structural studies also suggest a mechanism of reverse allostery that contributes to trapping, suggesting an additional mechanism of action for certain PARPi [46,152,153].

Originally PARPi have been shown to selectively affect BRCA1/2-deficient cells and they are now used for treatment of such cancers [154–156]. BRCA1/2 are not the only HR factors whose loss confers sensitivity to treatment. Indeed, studies demonstrate that Olaparib treatment can be effective in BRCA wild-type cancers [157]. This has been reflected in approvals for use of PARPi treatment in cancers that are platinum-sensitive, regardless of their BRCA status. Current research aims to better understand PARP signalling to further identify sensitivity markers for PARPi treatment in order to increase its therapeutic potential [158]. Indeed, the loss of some of the downstream factors of PARP1 signalling such as XRCC1 and ALC1 has already been demonstrated to sensitise cells to PARPi treatment [114,159,160] (Table 1); however, this has not been yet confirmed by any clinical trials to our knowledge. Interestingly, loss of PARP1 accessory factor HPF1 has also been observed to confer PARPi sensitivity [35,67,69]. It is important for autoPARylation of three serine residues which are key for PARP dissociation from the DNA [35]. Currently, it is unclear if HPF1 is a clinically relevant marker but it could serve as a potential target for combination therapy.

**Table 1:** Effects of changes in activity of ADPr regulators or binders on response to PARPi

Gene	Change	Effect	Clinical value	References
PARG	Loss of protein activity	Resistance	Can be targeted through ARH3 inhibition	(Gogola et al. [179]), (Gomez et al. [166])
ARH3	Loss of protein activity	Resistance	Can be targeted through PARGi inhibition	(Prokhorova et al. [71]), (Prokhorova et al. [35]), (Ipsen et al. [164])
PARP1	Loss or mutations of sites	Resistance	-	(Pettitt et al. [163]), (Ipsen et al. [164])
PARP2	Loss	Resistance	-	(Blessing et al. [165])
ALC1	Overexpression	Resistance	Possible marker	(Juhasz et al. [160])
HPF1	Loss	Sensitivity	Possible marker	(Gibbs-Seymour et al. [67]), (Prokhorova et al. [35])
ARH3	Overexpression	Sensitivity	Possible marker	(Prokhorova et al. [71])
ALC1	Loss of protein activity	Sensitivity	Possible marker	(Juhasz et al. [160]), (Hewitt et al. [114]), (Verma et al. [159])
XRCC1	Loss of protein activity	Sensitivity	Possible marker	(Demin et al. [180])

While the development of PARPi has been a big success, most tumours eventually develop resistance to the treatment. This can occur through multiple mechanisms, including increased drug efflux, the restoration of HR through BRCA reversion or the suppression of NHEJ [161,162]. As the toxicity of PARPi is mostly attributed to the trapping of PARPs, the loss of PARP1 and PARP2 is another major resistance mechanism [163–165]. Recently, it was suggested that modulation of other ADPr signalling enzymes, such as ALC1 could lead to changes in the response to PARPi and thus resistance [160]. Loss of PARG activity and resistance to PARPi has been observed in cells exposed to chronic doses of PARPi [164,166]. More recently, the loss of ARH3 was shown to confer PARPi resistance in cell line models [35,71,164].

Loss of PARG activity in response to treatment with PARPi makes ARH3 a possible target in tumours and ARH3 inhibitors are currently being developed for this purpose [71,121,167,168]. Conversely, in theory PARG inhibitors are another emerging treatment that can selectively target cells that lose ARH3 activity in response to PARPi [71,169]. They could also possibly be utilised as a primary therapy as it has been demonstrated that certain replication defects confer PARGi sensitivity [170–173]. The exact mechanism of this sensitivity is being elucidated and possible markers are being identified [174,175]. This is a promising avenue of cancer therapy that can hopefully fill the therapeutic gaps left by PARPi. PARG inhibitors have recently entered clinical trials (IDEAYA Biosciences).

## Future directions

The discovery of HPF1 and the importance of serine as a target of ADPr were significant advancements in our understanding of ADPr signalling in the DDR. However, many unanswered questions remain. What are the functional consequences of specific serine ADP-ribosylation modifications on hundreds of different proteins/sites? What is different about the glutamate ADP-ribosylation modifications? While we have a general picture of the canonical ADPr response, there are still a lot of details that remain elusive. Does DNA–ADPr occur in a physiological manner in mammalian cells or is it a pathological lesion [176]? There is also emerging evidence suggesting that other members of the PARP family are involved in the DDR [177,178]. Altogether, there is still a lot to uncover in the world of ADP-ribosylation. A variety of new protein players and potential drug targets are coming into view. Identification of new markers brings hope for expanding the therapeutic potential of already available PARPi treatments. Better understanding of PARPi resistance can aid clinicians in devising better treatment plans that address the issue. Since ADPr signalling is so fundamental to genome stability, targeting other factors in the pathway has potential to have therapeutic benefit. PARG inhibitors have recently entered clinical trials and ARH3 inhibitors are being developed. The clinical implications reach beyond cancer therapy as the role of ADPr in neurodegeneration is being better understood. The stage has been set for discovering the full picture of ADP-ribosylation signalling in DDR.

## Perspectives

- DNA repair PARPs act as sensors of DNA breaks and regulate different aspects of the DDR to maintain genome stability. PARPs are involved in the recruitment of DDR factors, chromatin remodelling and can mediate cell death upon overactivation.
- Our understanding of ADPr in the DDR has been expanded by the discovery of serine ADPr and the recent identification of HPF1 as an important factor that changes the specificity of PARP1/2 towards serine residues both on histones and PARPs. However, the exact mechanisms and roles of Ser-ADPr remain elusive and need to be further studied.
- It is emerging that modulation of ADP-ribosylation signalling can alter the efficacy of clinical PARPi therapy. Identification of biomarkers which would modulate PARPi sensitivity will be essential for efficient treatment with PARPi but development of new inhibitors for ADP-ribosylation factors eg. ARH3, ALC1, PARG or HPF1 could have big clinical implications.

## Open Access

Open access for this article was enabled by the participation of University of Oxford in an all-inclusive *Read & Publish* agreement with Portland Press and the Biochemical Society under a transformative agreement with JISC.

## Abbreviations

ADPr: ADP-ribosylation
AIF: apoptosis-inducing factor
APFL: Aprataxin-and-PNK-like factor
ARH3: (ADP-ribosyl)hydrolase 3
ART: ADP-ribose transferase
ATP: adenosine triphosphate
BER: Base Excision Repair
BRCA: Breast cancer gene
BRCT: BRCA1 C-terminal domain
CONDSIAS: childhood-onset neurodegeneration with variable ataxia and seizures
Cryo-EM: cryogenic electron microscopy
DDR: DNA damage response
FHA: forkhead-associated
HPF1: histone parylation factor 1
HR: homologous recombination
MAR: mono-ADP-ribose
MMEJ: microhomology-mediated end joining
MMS: methyl methanesulfonate
NER: nucleotide excision repair
NHEJ: non-homologous end joining
NTR: N-terminal region
PAR: poly-ADP-ribose
PARG: poly(ADP-ribose) glycohydrolase
PARP: poly(ADP-ribose) polymerase
PARPi: PARP inhibitors
PNKP: polynucleotide phosphatase/kinase
PTM: post-translational modification
SSB: single-stranded break
TARG1: terminal ADP-ribose glycohydrolase
XRCC1: X-ray repair cross-complementing protein 1

## References

[1] Blackford, A.N. and Jackson, S.P. (2017) ATM, ATR, and DNA-PK: the Trinity at the heart of the DNA damage response. Mol. Cell 66, 801–817 10.1016/j.molcel.2017.05.015 28622525

[2] Konopka, A. and Atkin, J.D. (2022) The role of DNA damage in neural plasticity in physiology and neurodegeneration. Front. Cell Neurosci. 16, 836885 10.3389/fncel.2022.836885 35813507 PMC9259845

[3] Luscher, B., Ahel, I., Altmeyer, M., Ashworth, A., Bai, P., Chang, P. et al. (2022) ADP-ribosyltransferases, an update on function and nomenclature. FEBS J. 289, 7399–7410 10.1111/febs.16142 34323016 PMC9027952

[4] Fontana, P., Bonfiglio, J.J., Palazzo, L., Bartlett, E., Matic, I. and Ahel, I. (2017) Serine ADP-ribosylation reversal by the hydrolase ARH3. eLife 6, e28533 10.7554/eLife.28533 28650317 PMC5552275

[5] Bonfiglio, J.J., Leidecker, O., Dauben, H., Longarini, E.J., Colby, T., San Segundo-Acosta, P. et al. (2020) An HPF1/PARP1-based chemical biology strategy for exploring ADP-ribosylation. Cell 183, 1086–102.e23 10.1016/j.cell.2020.09.055 33186521

[6] Palazzo, L., Leidecker, O., Prokhorova, E., Dauben, H., Matic, I. and Ahel, I. (2018) Serine is the major residue for ADP-ribosylation upon DNA damage. eLife 7, e34334 10.7554/eLife.34334 29480802 PMC5837557

[7] Vyas, S., Matic, I., Uchima, L., Rood, J., Zaja, R., Hay, R.T. et al. (2014) Family-wide analysis of poly(ADP-ribose) polymerase activity. Nat. Commun. 5, 4426 10.1038/ncomms5426 25043379 PMC4123609

[8] Bonfiglio, J.J., Fontana, P., Zhang, Q., Colby, T., Gibbs-Seymour, I., Atanassov, I. et al. (2017) Serine ADP-ribosylation depends on HPF1. Mol. Cell 65, 932–40.e6 10.1016/j.molcel.2017.01.003 28190768 PMC5344681

[9] Hendriks, I.A., Buch-Larsen, S.C., Prokhorova, E., Elsborg, J.D., Rebak, A., Zhu, K. et al. (2021) The regulatory landscape of the human HPF1- and ARH3-dependent ADP-ribosylome. Nat. Commun. 12, 5893 10.1038/s41467-021-26172-4 34625544 PMC8501107

[10] Rodriguez, K.M., Buch-Larsen, S.C., Kirby, I.T., Siordia, I.R., Hutin, D., Rasmussen, M. et al. (2021) Chemical genetics and proteome-wide site mapping reveal cysteine MARylation by PARP-7 on immune-relevant protein targets. eLife 10, e60480 10.7554/eLife.60480 33475084 PMC7880690

[11] Gagne, J.P., Ethier, C., Defoy, D., Bourassa, S., Langelier, M.F., Riccio, A.A. et al. (2015) Quantitative site-specific ADP-ribosylation profiling of DNA-dependent PARPs. DNA Repair (Amst) 30, 68–79 10.1016/j.dnarep.2015.02.004 25800440

[12] Zhang, Y., Wang, J., Ding, M. and Yu, Y. (2013) Site-specific characterization of the Asp- and Glu-ADP-ribosylated proteome. Nat. Methods. 10, 981–984 10.1038/nmeth.2603 23955771

[13] Leslie Pedrioli, D.M., Leutert, M., Bilan, V., Nowak, K., Gunasekera, K., Ferrari, E. et al. (2018) Comprehensive ADP-ribosylome analysis identifies tyrosine as an ADP-ribose acceptor site. EMBO Rep. 19, e45310 10.15252/embr.201745310 29954836 PMC6073207

[14] Buch-Larsen, S.C., Hendriks, I.A., Lodge, J.M., Rykaer, M., Furtwangler, B., Shishkova, E. et al. (2020) Mapping physiological ADP-ribosylation using activated ion electron transfer dissociation. Cell Rep. 32, 108176 10.1016/j.celrep.2020.108176 32966781 PMC7508052

[15] Bartlett, E., Bonfiglio, J.J., Prokhorova, E., Colby, T., Zobel, F., Ahel, I. et al. (2018) Interplay of histone marks with serine ADP-ribosylation. Cell Rep. 24, 3488–502.e5 10.1016/j.celrep.2018.08.092 30257210 PMC6172693

[16] Aberle, L., Kruger, A., Reber, J.M., Lippmann, M., Hufnagel, M., Schmalz, M. et al. (2020) PARP1 catalytic variants reveal branching and chain length-specific functions of poly(ADP-ribose) in cellular physiology and stress response. Nucleic Acids Res. 48, 10015–10033 10.1093/nar/gkaa590 32667640 PMC7544232

[17] Loffler, T., Kruger, A., Zirak, P., Winterhalder, M.J., Muller, A.L., Fischbach, A. et al. (2023) Influence of chain length and branching on poly(ADP-ribose)-protein interactions. Nucleic Acids Res. 51, 536–552 10.1093/nar/gkac1235 36625274 PMC9881148

[18] Miwa, M., Saikawa, N., Yamaizumi, Z., Nishimura, S. and Sugimura, T. (1979) Structure of poly(adenosine diphosphate ribose): identification of 2′-[1″-ribosyl-2″-(or 3″-)(1‴-ribosyl)]adenosine-5′,5″,5‴-tris(phosphate) as a branch linkage. Proc. Natl Acad. Sci. U.S.A. 76, 595–599 10.1073/pnas.76.2.595 218210 PMC382995

[19] Zhu, K., Suskiewicz, M.J., Hlousek-Kasun, A., Meudal, H., Mikoc, A., Aucagne, V. et al. (2022) DELTEX e3 ligases ubiquitylate ADP-ribosyl modification on protein substrates. Sci. Adv. 8, eadd4253 10.1126/sciadv.add4253 36197986 PMC7615817

[20] Challa, S., Stokes, M.S. and Kraus, W.L. (2021) MARTs and MARylation in the cytosol: biological functions, mechanisms of action, and therapeutic potential. Cells 10, 313 10.3390/cells10020313 33546365 PMC7913519

[21] Crawford, K., Oliver, P.L., Agnew, T., Hunn, B.H.M. and Ahel, I. (2021) Behavioural characterisation of Macrod1 and Macrod2 knockout mice. Cells 10, 368 10.3390/cells10020368 33578760 PMC7916507

[22] Cohen, M.S. and Chang, P. (2018) Insights into the biogenesis, function, and regulation of ADP-ribosylation. Nat. Chem. Biol. 14, 236–243 10.1038/nchembio.2568 29443986 PMC5922452

[23] Fehr, A.R., Singh, S.A., Kerr, C.M., Mukai, S., Higashi, H. and Aikawa, M. (2020) The impact of PARPs and ADP-ribosylation on inflammation and host-pathogen interactions. Genes Dev. 34, 341–359 10.1101/gad.334425.119 32029454 PMC7050484

[24] Hendriks, I.A., Larsen, S.C. and Nielsen, M.L. (2019) An advanced strategy for comprehensive profiling of ADP-ribosylation sites using mass spectrometry-based proteomics. Mol. Cell. Proteomics 18, 1010–1026 10.1074/mcp.TIR119.001315 30798302 PMC6495254

[25] Hanzlikova, H., Gittens, W., Krejcikova, K., Zeng, Z. and Caldecott, K.W. (2017) Overlapping roles for PARP1 and PARP2 in the recruitment of endogenous XRCC1 and PNKP into oxidized chromatin. Nucleic Acids Res. 45, 2546–2557 10.1093/nar/gkw1246 27965414 PMC5389470

[26] Martin-Hernandez, K., Rodriguez-Vargas, J.M., Schreiber, V. and Dantzer, F. (2017) Expanding functions of ADP-ribosylation in the maintenance of genome integrity. Semin. Cell Dev. Biol. 63, 92–101 10.1016/j.semcdb.2016.09.009 27670719

[27] D'Amours, D., Desnoyers, S., D'Silva, I. and Poirier, G.G. (1999) Poly(ADP-ribosyl)ation reactions in the regulation of nuclear functions. Biochem. J. 342, 249–268 PMID:10455009 PMC1220459

[28] Woodhouse, B.C. and Dianov, G.L. (2008) Poly ADP-ribose polymerase-1: an international molecule of mystery. DNA Repair (Amst) 7, 1077–1086 10.1016/j.dnarep.2008.03.009 18468963

[29] Ame, J.C., Spenlehauer, C. and de Murcia, G. (2004) The PARP superfamily. Bioessays 26, 882–893 10.1002/bies.20085 15273990

[30] Suskiewicz, M.J., Palazzo, L., Hughes, R. and Ahel, I. (2021) Progress and outlook in studying the substrate specificities of PARPs and related enzymes. FEBS J. 288, 2131–2142 10.1111/febs.15518 32785980

[31] Okayama, H., Edson, C.M., Fukushima, M., Ueda, K. and Hayaishi, O. (1977) Purification and properties of poly(adenosine diphosphate ribose) synthetase. J. Biol. Chem. 252, 7000–7005 PMID:198398

[32] Langelier, M.F., Planck, J.L., Roy, S. and Pascal, J.M. (2012) Structural basis for DNA damage-dependent poly(ADP-ribosyl)ation by human PARP-1. Science 336, 728–732 10.1126/science.1216338 22582261 PMC3532513

[33] Rudolph, J., Muthurajan, U.M., Palacio, M., Mahadevan, J., Roberts, G., Erbse, A.H. et al. (2021) The BRCT domain of PARP1 binds intact DNA and mediates intrastrand transfer. Mol. Cell 81, 4994–5006.e5 10.1016/j.molcel.2021.11.014 34919819 PMC8769213

[34] Zhang, N., Zhang, Y., Miao, W., Shi, C., Chen, Z., Wu, B. et al. (2022) An unexpected role for BAG3 in regulating PARP1 ubiquitination in oxidative stress-related endothelial damage. Redox Biol. 50, 102238 10.1016/j.redox.2022.102238 35066290 PMC8783151

[35] Prokhorova, E., Zobel, F., Smith, R., Zentout, S., Gibbs-Seymour, I., Schutzenhofer, K. et al. (2021) Serine-linked PARP1 auto-modification controls PARP inhibitor response. Nat. Commun. 12, 4055 10.1038/s41467-021-24361-9 34210965 PMC8249464

[36] Langelier, M.F., Eisemann, T., Riccio, A.A. and Pascal, J.M. (2018) PARP family enzymes: regulation and catalysis of the poly(ADP-ribose) posttranslational modification. Curr. Opin. Struct. Biol. 53, 187–198 10.1016/j.sbi.2018.11.002 30481609 PMC6687463

[37] Obaji, E., Haikarainen, T. and Lehtio, L. (2018) Structural basis for DNA break recognition by ARTD2/PARP2. Nucleic Acids Res. 46, 12154–12165 10.1093/nar/gky927 30321391 PMC6294510

[38] Eustermann, S., Wu, W.F., Langelier, M.F., Yang, J.C., Easton, L.E., Riccio, A.A. et al. (2015) Structural basis of detection and signaling of DNA single-strand breaks by human PARP-1. Mol. Cell 60, 742–754 10.1016/j.molcel.2015.10.032 26626479 PMC4678113

[39] Rouleau-Turcotte, E., Krastev, D.B., Pettitt, S.J., Lord, C.J. and Pascal, J.M. (2022) Captured snapshots of PARP1 in the active state reveal the mechanics of PARP1 allostery. Mol. Cell 82, 2939–51.e5 10.1016/j.molcel.2022.06.011 35793673 PMC9391306

[40] Langelier, M.F. and Pascal, J.M. (2013) PARP-1 mechanism for coupling DNA damage detection to poly(ADP-ribose) synthesis. Curr. Opin. Struct. Biol. 23, 134–143 10.1016/j.sbi.2013.01.003 23333033 PMC3572337

[41] Robu, M., Shah, R.G., Petitclerc, N., Brind'Amour, J., Kandan-Kulangara, F. and Shah, G.M. (2013) Role of poly(ADP-ribose) polymerase-1 in the removal of UV-induced DNA lesions by nucleotide excision repair. Proc. Natl Acad. Sci. U.S.A. 110, 1658–1663 10.1073/pnas.1209507110 23319653 PMC3562836

[42] Langelier, M.F., Planck, J.L., Roy, S. and Pascal, J.M. (2011) Crystal structures of poly(ADP-ribose) polymerase-1 (PARP-1) zinc fingers bound to DNA: structural and functional insights into DNA-dependent PARP-1 activity. J. Biol. Chem. 286, 10690–10701 10.1074/jbc.M110.202507 21233213 PMC3060520

[43] Ali, A.A.E., Timinszky, G., Arribas-Bosacoma, R., Kozlowski, M., Hassa, P.O., Hassler, M. et al. (2012) The zinc-finger domains of PARP1 cooperate to recognize DNA strand breaks. Nat. Struct. Mol. Biol. 19, 685–692 10.1038/nsmb.2335 22683995 PMC4826610

[44] Langelier, M.F., Riccio, A.A. and Pascal, J.M. (2014) PARP-2 and PARP-3 are selectively activated by 5′ phosphorylated DNA breaks through an allosteric regulatory mechanism shared with PARP-1. Nucleic Acids Res. 42, 7762–7775 10.1093/nar/gku474 24928857 PMC4081085

[45] Dawicki-McKenna, J.M., Langelier, M.F., DeNizio, J.E., Riccio, A.A., Cao, C.D., Karch, K.R. et al. (2015) PARP-1 activation requires local unfolding of an autoinhibitory domain. Mol. Cell 60, 755–768 10.1016/j.molcel.2015.10.013 26626480 PMC4712911

[46] Langelier, M.F., Zandarashvili, L., Aguiar, P.M., Black, B.E. and Pascal, J.M. (2018) NAD(+) analog reveals PARP-1 substrate-blocking mechanism and allosteric communication from catalytic center to DNA-binding domains. Nat. Commun. 9, 844 10.1038/s41467-018-03234-8 29487285 PMC5829251

[47] Caldecott, K.W. (2019) XRCC1 protein; form and function. DNA Repair (Amst) 81, 102664 10.1016/j.dnarep.2019.102664 31324530

[48] Ahel, D., Horejsi, Z., Wiechens, N., Polo, S.E., Garcia-Wilson, E., Ahel, I. et al. (2009) Poly(ADP-ribose)-dependent regulation of DNA repair by the chromatin remodeling enzyme ALC1. Science 325, 1240–1243 10.1126/science.1177321 19661379 PMC3443743

[49] Sellou, H., Lebeaupin, T., Chapuis, C., Smith, R., Hegele, A., Singh, H.R. et al. (2016) The poly(ADP-ribose)-dependent chromatin remodeler Alc1 induces local chromatin relaxation upon DNA damage. Mol. Biol. Cell 27, 3791–3799 10.1091/mbc.E16-05-0269 27733626 PMC5170603

[50] Mehrotra, P.V., Ahel, D., Ryan, D.P., Weston, R., Wiechens, N., Kraehenbuehl, R. et al. (2011) DNA repair factor APLF is a histone chaperone. Mol. Cell 41, 46–55 10.1016/j.molcel.2010.12.008 21211722 PMC3443741

[51] Posavec Marjanovic, M., Crawford, K. and Ahel, I. (2017) PARP, transcription and chromatin modeling. Semin. Cell Dev. Biol. 63, 102–113 10.1016/j.semcdb.2016.09.014 27677453

[52] Zong, W., Gong, Y., Sun, W., Li, T. and Wang, Z.Q. (2022) PARP1: liaison of chromatin remodeling and transcription. Cancers (Basel) 14, 4162 10.3390/cancers14174162 36077699 PMC9454564

[53] Kamaletdinova, T., Fanaei-Kahrani, Z. and Wang, Z.Q. (2019) The enigmatic function of PARP1: from PARylation activity to PAR readers. Cells 8, 1625 10.3390/cells8121625 31842403 PMC6953017

[54] Menissier de Murcia, J., Ricoul, M., Tartier, L., Niedergang, C., Huber, A., Dantzer, F. et al. (2003) Functional interaction between PARP-1 and PARP-2 in chromosome stability and embryonic development in mouse. EMBO J. 22, 2255–2263 10.1093/emboj/cdg206 12727891 PMC156078

[55] Riccio, A.A., Cingolani, G. and Pascal, J.M. (2016) PARP-2 domain requirements for DNA damage-dependent activation and localization to sites of DNA damage. Nucleic Acids Res. 44, 1691–1702 10.1093/nar/gkv1376 26704974 PMC4770219

[56] Chen, Q., Kassab, M.A., Dantzer, F. and Yu, X. (2018) PARP2 mediates branched poly ADP-ribosylation in response to DNA damage. Nat. Commun. 9, 3233 10.1038/s41467-018-05588-5 30104678 PMC6089979

[57] Liu, C., Vyas, A., Kassab, M.A., Singh, A.K. and Yu, X. (2017) The role of poly ADP-ribosylation in the first wave of DNA damage response. Nucleic Acids Res. 45, 8129–8141 10.1093/nar/gkx565 28854736 PMC5737498

[58] Gaullier, G., Roberts, G., Muthurajan, U.M., Bowerman, S., Rudolph, J., Mahadevan, J. et al. (2020) Bridging of nucleosome-proximal DNA double-strand breaks by PARP2 enhances its interaction with HPF1. PLoS ONE 15, e0240932 10.1371/journal.pone.0240932 33141820 PMC7608914

[59] Loseva, O., Jemth, A.S., Bryant, H.E., Schuler, H., Lehtio, L., Karlberg, T. et al. (2010) PARP-3 is a mono-ADP-ribosylase that activates PARP-1 in the absence of DNA. J. Biol. Chem. 285, 8054–8060 10.1074/jbc.M109.077834 20064938 PMC2832956

[60] Grundy, G.J., Polo, L.M., Zeng, Z., Rulten, S.L., Hoch, N.C., Paomephan, P. et al. (2016) PARP3 is a sensor of nicked nucleosomes and monoribosylates histone H2B(Glu2). Nat. Commun. 7, 12404 10.1038/ncomms12404 27530147 PMC4992063

[61] Rulten, S.L., Fisher, A.E., Robert, I., Zuma, M.C., Rouleau, M., Ju, L. et al. (2011) PARP-3 and APLF function together to accelerate nonhomologous end-joining. Mol. Cell 41, 33–45 10.1016/j.molcel.2010.12.006 21211721

[62] Rouleau, M., Saxena, V., Rodrigue, A., Paquet, E.R., Gagnon, A., Hendzel, M.J. et al. (2011) A key role for poly(ADP-ribose) polymerase 3 in ectodermal specification and neural crest development. PLoS ONE 6, e15834 10.1371/journal.pone.0015834 21264220 PMC3022025

[63] Beck, C., Rodriguez-Vargas, J.M., Boehler, C., Robert, I., Heyer, V., Hanini, N. et al. (2019) PARP3, a new therapeutic target to alter Rictor/mTORC2 signaling and tumor progression in BRCA1-associated cancers. Cell Death Differ. 26, 1615–1630 10.1038/s41418-018-0233-1 30442946 PMC6748154

[64] Boehler, C., Gauthier, L.R., Mortusewicz, O., Biard, D.S., Saliou, J.M., Bresson, A. et al. (2011) Poly(ADP-ribose) polymerase 3 (PARP3), a newcomer in cellular response to DNA damage and mitotic progression. Proc. Natl Acad. Sci. U.S.A. 108, 2783–2788 10.1073/pnas.1016574108 21270334 PMC3041075

[65] Hakme, A., Wong, H.K., Dantzer, F. and Schreiber, V. (2008) The expanding field of poly(ADP-ribosyl)ation reactions. ‘protein modifications: beyond the usual suspects’ review series. EMBO Rep. 9, 1094–1100 10.1038/embor.2008.191 18927583 PMC2581850

[66] Crawford, K., Bonfiglio, J.J., Mikoc, A., Matic, I. and Ahel, I. (2018) Specificity of reversible ADP-ribosylation and regulation of cellular processes. Crit. Rev. Biochem. Mol. Biol. 53, 64–82 10.1080/10409238.2017.1394265 29098880

[67] Gibbs-Seymour, I., Fontana, P., Rack, J.G.M. and Ahel, I. (2016) HPF1/C4orf27 is a PARP-1-interacting protein that regulates PARP-1 ADP-ribosylation activity. Mol. Cell 62, 432–442 10.1016/j.molcel.2016.03.008 27067600 PMC4858568

[68] Leidecker, O., Bonfiglio, J.J., Colby, T., Zhang, Q., Atanassov, I., Zaja, R. et al. (2016) Serine is a new target residue for endogenous ADP-ribosylation on histones. Nat. Chem. Biol. 12, 998–1000 10.1038/nchembio.2180 27723750 PMC5113755

[69] Suskiewicz, M.J., Zobel, F., Ogden, T.E.H., Fontana, P., Ariza, A., Yang, J.C. et al. (2020) HPF1 completes the PARP active site for DNA damage-induced ADP-ribosylation. Nature 579, 598–602 10.1038/s41586-020-2013-6 32028527 PMC7104379

[70] Sun, F.H., Zhao, P., Zhang, N., Kong, L.L., Wong, C.C.L. and Yun, C.H. (2021) HPF1 remodels the active site of PARP1 to enable the serine ADP-ribosylation of histones. Nat. Commun. 12, 1028 10.1038/s41467-021-21302-4 33589610 PMC7884425

[71] Prokhorova, E., Agnew, T., Wondisford, A.R., Tellier, M., Kaminski, N., Beijer, D. et al. (2021) Unrestrained poly-ADP-ribosylation provides insights into chromatin regulation and human disease. Mol. Cell 81, 2640–55.e8 10.1016/j.molcel.2021.04.028 34019811 PMC8221567

[72] Langelier, M.F., Billur, R., Sverzhinsky, A., Black, B.E. and Pascal, J.M. (2021) HPF1 dynamically controls the PARP1/2 balance between initiating and elongating ADP-ribose modifications. Nat. Commun. 12, 6675 10.1038/s41467-021-27043-8 34795260 PMC8602370

[73] Smith, R., Zentout, S., Rother, M., Bigot, N., Chapuis, C., Mihut´, A. et al. (2023) HPF1-dependent histone ADP-ribosylation triggers chromatin relaxation to promote the recruitment of repair factors at sites of DNA damage. Nat. Struct. Mol. Biol. 10.1038/s41594-023-00977-x 37106138

[74] Rudolph, J., Roberts, G., Muthurajan, U.M. and Luger, K. (2021) HPF1 and nucleosomes mediate a dramatic switch in activity of PARP1 from polymerase to hydrolase. eLife 10, e65773 10.7554/eLife.65773 33683197 PMC8012059

[75] Larsen, S.C., Hendriks, I.A., Lyon, D., Jensen, L.J. and Nielsen, M.L. (2018) Systems-wide analysis of serine ADP-ribosylation reveals widespread occurrence and site-specific overlap with phosphorylation. Cell Rep. 24, 2493–505.e4 10.1016/j.celrep.2018.07.083 30157440

[76] Murai, J., Huang, S.Y., Das, B.B., Renaud, A., Zhang, Y., Doroshow, J.H. et al. (2012) Trapping of PARP1 and PARP2 by clinical PARP inhibitors. Cancer Res. 72, 5588–5599 10.1158/0008-5472.CAN-12-2753 23118055 PMC3528345

[77] Murai, J. and Pommier, Y. (2019) PARP trapping beyond homologous recombination and platinum sensitivity in cancers. Annu. Rev. Cancer Biol. 3, 131–150 10.1146/annurev-cancerbio-030518-055914

[78] Krastev, D.B., Wicks, A.J. and Lord, C.J. (2021) PARP inhibitors: trapped in a toxic love affair. Cancer Res. 81, 5605–5607 10.1158/0008-5472.CAN-21-3201 34782321

[79] Schoonen, P.M., Talens, F., Stok, C., Gogola, E., Heijink, A.M., Bouwman, P. et al. (2017) Progression through mitosis promotes PARP inhibitor-induced cytotoxicity in homologous recombination-deficient cancer cells. Nat. Commun. 8, 15981 10.1038/ncomms15981 28714471 PMC5520019

[80] Onji, H. and Murai, J. (2022) Reconsidering the mechanisms of action of PARP inhibitors based on clinical outcomes. Cancer Sci. 113, 2943–2951 10.1111/cas.15477 35766436 PMC9459283

[81] Palazzo, L., Suskiewicz, M.J. and Ahel, I. (2021) Serine ADP-ribosylation in DNA-damage response regulation. Curr. Opin. Genet. Dev. 71, 106–113 10.1016/j.gde.2021.07.005 34340015

[82] Kumamoto, S., Nishiyama, A., Chiba, Y., Miyashita, R., Konishi, C., Azuma, Y. et al. (2021) HPF1-dependent PARP activation promotes LIG3-XRCC1-mediated backup pathway of Okazaki fragment ligation. Nucleic Acids Res. 49, 5003–5016 10.1093/nar/gkab269 33872376 PMC8136790

[83] Mosler, T., Baymaz, H.I., Graf, J.F., Mikicic, I., Blattner, G., Bartlett, E. et al. (2022) PARP1 proximity proteomics reveals interaction partners at stressed replication forks. Nucleic Acids Res. 50, 11600–11618 10.1093/nar/gkac948 36350633 PMC9723622

[84] Genois, M.M., Gagne, J.P., Yasuhara, T., Jackson, J., Saxena, S., Langelier, M.F. et al. (2021) CARM1 regulates replication fork speed and stress response by stimulating PARP1. Mol. Cell 81, 784–800.e8 10.1016/j.molcel.2020.12.010 33412112 PMC7897296

[85] Munnur, D., Bartlett, E., Mikolcevic, P., Kirby, I.T., Rack, J.G.M., Mikoc, A. et al. (2019) Reversible ADP-ribosylation of RNA. Nucleic Acids Res. 47, 5658–5669 10.1093/nar/gkz305 31216043 PMC6582358

[86] Groslambert, J., Prokhorova, E. and Ahel, I. (2021) ADP-ribosylation of DNA and RNA. DNA Repair (Amst) 105, 103144 10.1016/j.dnarep.2021.103144 34116477 PMC8385414

[87] Munnur, D. and Ahel, I. (2017) Reversible mono-ADP-ribosylation of DNA breaks. FEBS J. 284, 4002–4016 10.1111/febs.14297 29054115 PMC5725667

[88] Talhaoui, I., Lebedeva, N.A., Zarkovic, G., Saint-Pierre, C., Kutuzov, M.M., Sukhanova, M.V. et al. (2016) Poly(ADP-ribose) polymerases covalently modify strand break termini in DNA fragments in vitro. Nucleic Acids Res. 44, 9279–9295 10.1093/nar/gkw675 27471034 PMC5100588

[89] Zarkovic, G., Belousova, E.A., Talhaoui, I., Saint-Pierre, C., Kutuzov, M.M., Matkarimov, B.T. et al. (2018) Characterization of DNA ADP-ribosyltransferase activities of PARP2 and PARP3: new insights into DNA ADP-ribosylation. Nucleic Acids Res. 46, 2417–2431 10.1093/nar/gkx1318 29361132 PMC5861426

[90] Matta, E., Kiribayeva, A., Khassenov, B., Matkarimov, B.T. and Ishchenko, A.A. (2020) Insight into DNA substrate specificity of PARP1-catalysed DNA poly(ADP-ribosyl)ation. Sci. Rep. 10, 3699 10.1038/s41598-020-60631-0 32111879 PMC7048826

[91] Belousova, E.A., Ishchenko, A.A. and Lavrik, O.I. (2018) Dna is a new target of Parp3. Sci. Rep. 8, 4176 10.1038/s41598-018-22673-3 29520010 PMC5843604

[92] Musheev, M.U., Schomacher, L., Basu, A., Han, D., Krebs, L., Scholz, C. et al. (2022) Mammalian N1-adenosine PARylation is a reversible DNA modification. Nat. Commun. 13, 6138 10.1038/s41467-022-33731-w 36253381 PMC9576699

[93] Tromans-Coia, C., Sanchi, A., Moeller, G.K., Timinszky, G., Lopes, M. and Ahel, I. (2021) TARG1 protects against toxic DNA ADP-ribosylation. Nucleic Acids Res. 49, 10477–10492 10.1093/nar/gkab771 34508355 PMC8501950

[94] Schuller, M., Butler, R.E., Ariza, A., Tromans-Coia, C., Jankevicius, G., Claridge, T.D.W. et al. (2021) Molecular basis for DarT ADP-ribosylation of a DNA base. Nature 596, 597–602 10.1038/s41586-021-03825-4 34408320

[95] Rack, J.G., Perina, D. and Ahel, I. (2016) Macrodomains: structure, function, evolution, and catalytic activities. Annu. Rev. Biochem. 85, 431–454 10.1146/annurev-biochem-060815-014935 26844395

[96] Teloni, F. and Altmeyer, M. (2016) Readers of poly(ADP-ribose): designed to be fit for purpose. Nucleic Acids Res. 44, 993–1006 10.1093/nar/gkv1383 26673700 PMC4756826

[97] Li, M., Lu, L.Y., Yang, C.Y., Wang, S. and Yu, X. (2013) The FHA and BRCT domains recognize ADP-ribosylation during DNA damage response. Genes Dev. 27, 1752–1768 10.1101/gad.226357.113 23964092 PMC3759693

[98] London, R.E. (2015) The structural basis of XRCC1-mediated DNA repair. DNA Repair (Amst) 30, 90–103 10.1016/j.dnarep.2015.02.005 25795425 PMC5580684

[99] Caldecott, K.W., McKeown, C.K., Tucker, J.D., Ljungquist, S. and Thompson, L.H. (1994) An interaction between the mammalian DNA repair protein XRCC1 and DNA ligase III. Mol. Cell. Biol. 14, 68–76 10.1128/mcb.14.1.68-76.1994 8264637 PMC358357

[100] Hammel, M., Rashid, I., Sverzhinsky, A., Pourfarjam, Y., Tsai, M.S., Ellenberger, T. et al. (2021) An atypical BRCT-BRCT interaction with the XRCC1 scaffold protein compacts human DNA ligase IIIalpha within a flexible DNA repair complex. Nucleic Acids Res. 49, 306–321 10.1093/nar/gkaa1188 33330937 PMC7797052

[101] Marintchev, A., Robertson, A., Dimitriadis, E.K., Prasad, R., Wilson, S.H. and Mullen, G.P. (2000) Domain specific interaction in the XRCC1-DNA polymerase beta complex. Nucleic Acids Res. 28, 2049–2059 10.1093/nar/28.10.2049 10773072 PMC105377

[102] Breslin, C., Hornyak, P., Ridley, A., Rulten, S.L., Hanzlikova, H., Oliver, A.W. et al. (2015) The XRCC1 phosphate-binding pocket binds poly (ADP-ribose) and is required for XRCC1 function. Nucleic Acids Res. 43, 6934–6944 10.1093/nar/gkv623 26130715 PMC4538820

[103] Polo, L.M., Xu, Y., Hornyak, P., Garces, F., Zeng, Z., Hailstone, R. et al. (2019) Efficient single-strand break repair requires binding to both poly(ADP-Ribose) and DNA by the central BRCT domain of XRCC1. Cell Rep. 26, 573–81.e5 10.1016/j.celrep.2018.12.082 30650352 PMC6334254

[104] El-Khamisy, S.F., Masutani, M., Suzuki, H. and Caldecott, K.W. (2003) A requirement for PARP-1 for the assembly or stability of XRCC1 nuclear foci at sites of oxidative DNA damage. Nucleic Acids Res. 31, 5526–5533 10.1093/nar/gkg761 14500814 PMC206461

[105] Ahel, I., Ahel, D., Matsusaka, T., Clark, A.J., Pines, J., Boulton, S.J. et al. (2008) Poly(ADP-ribose)-binding zinc finger motifs in DNA repair/checkpoint proteins. Nature 451, 81–85 10.1038/nature06420 18172500

[106] Rulten, S.L., Cortes-Ledesma, F., Guo, L., Iles, N.J. and Caldecott, K.W. (2008) APLF (C2orf13) is a novel component of poly(ADP-ribose) signaling in mammalian cells. Mol. Cell. Biol. 28, 4620–4628 10.1128/MCB.02243-07 18474613 PMC2447129

[107] Grundy, G.J., Rulten, S.L., Zeng, Z., Arribas-Bosacoma, R., Iles, N., Manley, K. et al. (2013) APLF promotes the assembly and activity of non-homologous end joining protein complexes. EMBO J. 32, 112–125 10.1038/emboj.2012.304 23178593 PMC3545299

[108] Corbeski, I., Dolinar, K., Wienk, H., Boelens, R. and van Ingen, H. (2018) DNA repair factor APLF acts as a H2A-H2B histone chaperone through binding its DNA interaction surface. Nucleic Acids Res. 46, 7138–7152 10.1093/nar/gky507 29905837 PMC6101569

[109] Corbeski, I., Guo, X., Eckhardt, B.V., Fasci, D., Wiegant, W., Graewert, M.A. et al. (2022) Chaperoning of the histone octamer by the acidic domain of DNA repair factor APLF. Sci. Adv. 8, eabo0517 10.1126/sciadv.abo0517 35895815 PMC9328677

[110] Pines, A., Vrouwe, M.G., Marteijn, J.A., Typas, D., Luijsterburg, M.S., Cansoy, M. et al. (2012) PARP1 promotes nucleotide excision repair through DDB2 stabilization and recruitment of ALC1. J. Cell Biol. 199, 235–249 10.1083/jcb.201112132 23045548 PMC3471223

[111] Gottschalk, A.J., Timinszky, G., Kong, S.E., Jin, J., Cai, Y., Swanson, S.K. et al. (2009) Poly(ADP-ribosyl)ation directs recruitment and activation of an ATP-dependent chromatin remodeler. Proc. Natl Acad. Sci. U.S.A. 106, 13770–4 10.1073/pnas.0906920106 19666485 PMC2722505

[112] Bacic, L., Gaullier, G., Sabantsev, A., Lehmann, L.C., Brackmann, K., Dimakou, D. et al. (2021) Structure and dynamics of the chromatin remodeler ALC1 bound to a PARylated nucleosome. eLife 10, e71420 10.7554/eLife.71420 34486521 PMC8463071

[113] Mohapatra, J., Tashiro, K., Beckner, R.L., Sierra, J., Kilgore, J.A., Williams, N.S. et al. (2021) Serine ADP-ribosylation marks nucleosomes for ALC1-dependent chromatin remodeling. eLife 10, e71502 10.7554/eLife.71502 34874266 PMC8683085

[114] Hewitt, G., Borel, V., Segura-Bayona, S., Takaki, T., Ruis, P., Bellelli, R. et al. (2021) Defective ALC1 nucleosome remodeling confers PARPi sensitization and synthetic lethality with HRD. Mol. Cell 81, 767–83.e11 10.1016/j.molcel.2020.12.006 33333017 PMC7895907

[115] Cheng, W., Su, Y. and Xu, F. (2013) CHD1L: a novel oncogene. Mol. Cancer 12, 170 10.1186/1476-4598-12-170 24359616 PMC3931672

[116] Kliza, K.W., Liu, Q., Roosenboom, L.W.M., Jansen, P., Filippov, D.V. and Vermeulen, M. (2021) Reading ADP-ribosylation signaling using chemical biology and interaction proteomics. Mol. Cell 81, 4552–67.e8 10.1016/j.molcel.2021.08.037 34551281

[117] Rack, J.G.M., Liu, Q., Zorzini, V., Voorneveld, J., Ariza, A., Honarmand Ebrahimi, K. et al. (2021) Mechanistic insights into the three steps of poly(ADP-ribosylation) reversal. Nat. Commun. 12, 4581 10.1038/s41467-021-24723-3 34321462 PMC8319183

[118] Tanuma, S. (1989) Evidence for a novel metabolic pathway of (ADP-ribose)n: pyrophosphorolysis of ADP-ribose in HeLa S3 cell nuclei. Biochem. Biophys. Res. Commun. 163, 1047–1055 10.1016/0006-291x(89)92327-9 2551267

[119] Oei, S.L. and Ziegler, M. (2000) ATP for the DNA ligation step in base excision repair is generated from poly(ADP-ribose). J. Biol. Chem. 275, 23234–9 10.1074/jbc.m002429200 10930429

[120] Wright, R.H., Lioutas, A., Le Dily, F., Soronellas, D., Pohl, A., Bonet, J. et al. (2016) ADP-ribose-derived nuclear ATP synthesis by NUDIX5 is required for chromatin remodeling. Science 352, 1221–1225 10.1126/science.aad9335 27257257

[121] Rack, J.G.M., Palazzo, L. and Ahel, I. (2020) (ADP-ribosyl)hydrolases: structure, function, and biology. Genes Dev. 34, 263–284 10.1101/gad.334631.119 32029451 PMC7050489

[122] Slade, D., Dunstan, M.S., Barkauskaite, E., Weston, R., Lafite, P., Dixon, N. et al. (2011) The structure and catalytic mechanism of a poly(ADP-ribose) glycohydrolase. Nature 477, 616–620 10.1038/nature10404 21892188 PMC3184140

[123] Dunstan, M.S., Barkauskaite, E., Lafite, P., Knezevic, C.E., Brassington, A., Ahel, M. et al. (2012) Structure and mechanism of a canonical poly(ADP-ribose) glycohydrolase. Nat. Commun. 3, 878 10.1038/ncomms1889 22673905

[124] Tucker, J.A., Bennett, N., Brassington, C., Durant, S.T., Hassall, G., Holdgate, G. et al. (2012) Structures of the human poly (ADP-ribose) glycohydrolase catalytic domain confirm catalytic mechanism and explain inhibition by ADP-HPD derivatives. PLoS ONE 7, e50889 10.1371/journal.pone.0050889 23251397 PMC3519477

[125] Barkauskaite, E., Brassington, A., Tan, E.S., Warwicker, J., Dunstan, M.S., Banos, B. et al. (2013) Visualization of poly(ADP-ribose) bound to PARG reveals inherent balance between exo- and endo-glycohydrolase activities. Nat. Commun. 4, 2164 10.1038/ncomms3164 23917065 PMC3741636

[126] Pourfarjam, Y., Ventura, J., Kurinov, I., Cho, A., Moss, J. and Kim, I.K. (2018) Structure of human ADP-ribosyl-acceptor hydrolase 3 bound to ADP-ribose reveals a conformational switch that enables specific substrate recognition. J. Biol. Chem. 293, 12350–9 10.1074/jbc.RA118.003586 29907568 PMC6093245

[127] Rack, J.G.M., Ariza, A., Drown, B.S., Henfrey, C., Bartlett, E., Shirai, T. et al. (2018) (ADP-ribosyl)hydrolases: structural basis for differential substrate recognition and inhibition. Cell Chem. Biol. 25, 1533–46.e12 10.1016/j.chembiol.2018.11.001 30472116 PMC6309922

[128] Oka, S., Kato, J. and Moss, J. (2006) Identification and characterization of a mammalian 39-kDa poly(ADP-ribose) glycohydrolase. J. Biol. Chem. 281, 705–713 10.1074/jbc.M510290200 16278211

[129] Sharifi, R., Morra, R., Appel, C.D., Tallis, M., Chioza, B., Jankevicius, G. et al. (2013) Deficiency of terminal ADP-ribose protein glycohydrolase TARG1/C6orf130 in neurodegenerative disease. EMBO J. 32, 1225–1237 10.1038/emboj.2013.51 23481255 PMC3642678

[130] Jankevicius, G., Hassler, M., Golia, B., Rybin, V., Zacharias, M., Timinszky, G. et al. (2013) A family of macrodomain proteins reverses cellular mono-ADP-ribosylation. Nat. Struct. Mol. Biol. 20, 508–514 10.1038/nsmb.2523 23474712 PMC7097781

[131] Rosenthal, F., Feijs, K.L., Frugier, E., Bonalli, M., Forst, A.H., Imhof, R. et al. (2013) Macrodomain-containing proteins are new mono-ADP-ribosylhydrolases. Nat. Struct. Mol. Biol. 20, 502–507 10.1038/nsmb.2521 23474714

[132] Hanai, S., Kanai, M., Ohashi, S., Okamoto, K., Yamada, M., Takahashi, H. et al. (2004) Loss of poly(ADP-ribose) glycohydrolase causes progressive neurodegeneration in Drosophila melanogaster. Proc. Natl Acad. Sci. U.S.A. 101, 82–86 10.1073/pnas.2237114100 14676324 PMC314142

[133] Koh, D.W., Lawler, A.M., Poitras, M.F., Sasaki, M., Wattler, S., Nehls, M.C. et al. (2004) Failure to degrade poly(ADP-ribose) causes increased sensitivity to cytotoxicity and early embryonic lethality. Proc. Natl Acad. Sci. U.S.A. 101, 17699–17704 10.1073/pnas.0406182101 15591342 PMC539714

[134] Beijer, D., Agnew, T., Rack, J.G.M., Prokhorova, E., Deconinck, T., Ceulemans, B. et al. (2021) Biallelic ADPRHL2 mutations in complex neuropathy affect ADP ribosylation and DNA damage response. Life Sci. Alliance 4, e202101057 10.26508/lsa.202101057 34479984 PMC8424258

[135] Hanzlikova, H., Prokhorova, E., Krejcikova, K., Cihlarova, Z., Kalasova, I., Kubovciak, J. et al. (2020) Pathogenic ARH3 mutations result in ADP-ribose chromatin scars during DNA strand break repair. Nat. Commun. 11, 3391 10.1038/s41467-020-17069-9 32636369 PMC7341855

[136] Danhauser, K., Alhaddad, B., Makowski, C., Piekutowska-Abramczuk, D., Syrbe, S., Gomez-Ospina, N. et al. (2018) Bi-allelic ADPRHL2 mutations cause neurodegeneration with developmental delay, ataxia, and axonal neuropathy. Am. J. Hum. Genet. 103, 817–825 10.1016/j.ajhg.2018.10.005 30401461 PMC6218634

[137] Ghosh, S.G., Becker, K., Huang, H., Dixon-Salazar, T., Chai, G., Salpietro, V. et al. (2018) Biallelic mutations in ADPRHL2, encoding ADP-ribosylhydrolase 3, lead to a degenerative pediatric stress-induced epileptic ataxia syndrome. Am. J. Hum. Genet. 103, 431–439 10.1016/j.ajhg.2018.07.010 30100084 PMC6128219

[138] Mashimo, M., Onishi, M., Uno, A., Tanimichi, A., Nobeyama, A., Mori, M. et al. (2021) The 89-kDa PARP1 cleavage fragment serves as a cytoplasmic PAR carrier to induce AIF-mediated apoptosis. J. Biol. Chem. 296, 100046 10.1074/jbc.RA120.014479 33168626 PMC7948984

[139] Mashimo, M., Kato, J. and Moss, J. (2013) ADP-ribosyl-acceptor hydrolase 3 regulates poly (ADP-ribose) degradation and cell death during oxidative stress. Proc. Natl Acad. Sci. U.S.A. 110, 18964–9 10.1073/pnas.1312783110 24191052 PMC3839768

[140] Liu, L., Li, J., Ke, Y., Zeng, X., Gao, J., Ba, X. et al. (2022) The key players of parthanatos: opportunities for targeting multiple levels in the therapy of parthanatos-based pathogenesis. Cell. Mol. Life Sci. 79, 60 10.1007/s00018-021-04109-w 35000037 PMC11073082

[141] Yu, S.W., Wang, H., Poitras, M.F., Coombs, C., Bowers, W.J., Federoff, H.J. et al. (2002) Mediation of poly(ADP-ribose) polymerase-1-dependent cell death by apoptosis-inducing factor. Science 297, 259–263 10.1126/science.1072221 12114629

[142] Niere, M., Kernstock, S., Koch-Nolte, F. and Ziegler, M. (2008) Functional localization of two poly(ADP-ribose)-degrading enzymes to the mitochondrial matrix. Mol. Cell. Biol. 28, 814–824 10.1128/MCB.01766-07 17991898 PMC2223433

[143] Farmer, H., McCabe, N., Lord, C.J., Tutt, A.N., Johnson, D.A., Richardson, T.B. et al. (2005) Targeting the DNA repair defect in BRCA mutant cells as a therapeutic strategy. Nature 434, 917–921 10.1038/nature03445 15829967

[144] Bryant, H.E., Schultz, N., Thomas, H.D., Parker, K.M., Flower, D., Lopez, E. et al. (2005) Specific killing of BRCA2-deficient tumours with inhibitors of poly(ADP-ribose) polymerase. Nature 434, 913–917 10.1038/nature03443 15829966

[145] Hay, T., Matthews, J.R., Pietzka, L., Lau, A., Cranston, A., Nygren, A.O. et al. (2009) Poly(ADP-ribose) polymerase-1 inhibitor treatment regresses autochthonous Brca2/p53-mutant mammary tumors *in vivo* and delays tumor relapse in combination with carboplatin. Cancer Res. 69, 3850–3855 10.1158/0008-5472.CAN-08-2388 19383921

[146] Fong, P.C., Boss, D.S., Yap, T.A., Tutt, A., Wu, P., Mergui-Roelvink, M. et al. (2009) Inhibition of poly(ADP-ribose) polymerase in tumors from BRCA mutation carriers. N. Engl. J. Med. 361, 123–134 10.1056/NEJMoa0900212 19553641

[147] Illuzzi, G., Staniszewska, A.D., Gill, S.J., Pike, A., McWilliams, L., Critchlow, S.E. et al. (2022) Preclinical characterization of AZD5305, a next-generation, highly selective PARP1 inhibitor and trapper. Clin. Cancer Res. 28, 4724–4736 10.1158/1078-0432.CCR-22-0301 35929986 PMC9623235

[148] Grimaldi, G. and Corda, D. (2019) ADP-ribosylation and intracellular traffic: an emerging role for PARP enzymes. Biochem. Soc. Trans. 47, 357–370 10.1042/BST20180416 30710058

[149] Strom, C.E., Johansson, F., Uhlen, M., Szigyarto, C.A., Erixon, K. and Helleday, T. (2011) Poly (ADP-ribose) polymerase (PARP) is not involved in base excision repair but PARP inhibition traps a single-strand intermediate. Nucleic Acids Res. 39, 3166–3175 10.1093/nar/gkq1241 21183466 PMC3082910

[150] Murai, J., Huang, S.Y., Renaud, A., Zhang, Y., Ji, J., Takeda, S. et al. (2014) Stereospecific PARP trapping by BMN 673 and comparison with olaparib and rucaparib. Mol. Cancer Ther. 13, 433–443 10.1158/1535-7163.MCT-13-0803 24356813 PMC3946062

[151] Kedar, P.S., Stefanick, D.F., Horton, J.K. and Wilson, S.H. (2012) Increased PARP-1 association with DNA in alkylation damaged, PARP-inhibited mouse fibroblasts. Mol. Cancer Res. 10, 360–368 10.1158/1541-7786.MCR-11-0477 22246237 PMC3307909

[152] Zandarashvili, L., Langelier, M.F., Velagapudi, U.K., Hancock, M.A., Steffen, J.D., Billur, R. et al. (2020) Structural basis for allosteric PARP-1 retention on DNA breaks. Science 368, eaax6367 10.1126/science.aax6367 32241924 PMC7347020

[153] Rudolph, J., Jung, K. and Luger, K. (2022) Inhibitors of PARP: number crunching and structure gazing. Proc. Natl Acad. Sci. U.S.A. 119, e2121979119 10.1073/pnas.2121979119 35259019 PMC8931346

[154] Kim, G., Ison, G., McKee, A.E., Zhang, H., Tang, S., Gwise, T. et al. (2015) FDA approval summary: olaparib monotherapy in patients with deleterious germline BRCA-mutated advanced ovarian cancer treated with three or more lines of chemotherapy. Clin. Cancer Res. 21, 4257–4261 10.1158/1078-0432.CCR-15-0887 26187614

[155] Robson, M., Im, S.A., Senkus, E., Xu, B., Domchek, S.M., Masuda, N. et al. (2017) Olaparib for metastatic breast cancer in patients with a germline BRCA mutation. N. Engl. J. Med. 377, 523–533 10.1056/NEJMoa1706450 28578601

[156] Anscher, M.S., Chang, E., Gao, X., Gong, Y., Weinstock, C., Bloomquist, E. et al. (2021) Fda approval summary: rucaparib for the treatment of patients with deleterious BRCA-mutated metastatic castrate-resistant prostate cancer. Oncologist 26, 139–146 10.1002/onco.13585 33145877 PMC7873319

[157] Ledermann, J.A. and Pujade-Lauraine, E. (2019) Olaparib as maintenance treatment for patients with platinum-sensitive relapsed ovarian cancer. Ther. Adv. Med. Oncol. 11, 1758835919849753 10.1177/1758835919849753 31205507 PMC6535754

[158] Awwad, S.W., Serrano-Benitez, A., Thomas, J.C., Gupta, V. and Jackson, S.P. (2023) Revolutionizing DNA repair research and cancer therapy with CRISPR-Cas screens. Nat. Rev. Mol. Cell Biol. 10.1038/s41580-022-00571-x 36781955

[159] Verma, P., Zhou, Y., Cao, Z., Deraska, P.V., Deb, M., Arai, E. et al. (2021) ALC1 links chromatin accessibility to PARP inhibitor response in homologous recombination-deficient cells. Nat. Cell Biol. 23, 160–171 10.1038/s41556-020-00624-3 33462394 PMC7880902

[160] Juhasz, S., Smith, R., Schauer, T., Spekhardt, D., Mamar, H., Zentout, S. et al. (2020) The chromatin remodeler ALC1 underlies resistance to PARP inhibitor treatment. Sci. Adv. 6, eabb8626 10.1126/sciadv.abb8626 33355125 PMC11206534

[161] Fugger, K., Hewitt, G., West, S.C. and Boulton, S.J. (2021) Tackling PARP inhibitor resistance. Trends Cancer 7, 1102–1118 10.1016/j.trecan.2021.08.007 34563478

[162] Washington, C.R. and Moore, K.N. (2022) Resistance to poly (ADP-Ribose) polymerase inhibitors (PARPi): mechanisms and potential to reverse. Curr. Oncol. Rep. 24, 1685–1693 10.1007/s11912-022-01337-6 36346509

[163] Pettitt, S.J., Krastev, D.B., Brandsma, I., Drean, A., Song, F., Aleksandrov, R. et al. (2018) Genome-wide and high-density CRISPR-Cas9 screens identify point mutations in PARP1 causing PARP inhibitor resistance. Nat. Commun. 9, 1849 10.1038/s41467-018-03917-2 29748565 PMC5945626

[164] Ipsen, M.B., Sorensen, E.M.G., Thomsen, E.A., Weiss, S., Haldrup, J., Dalby, A. et al. (2022) A genome-wide CRISPR-Cas9 knockout screen identifies novel PARP inhibitor resistance genes in prostate cancer. Oncogene 41, 4271–4281 10.1038/s41388-022-02427-2 35933519

[165] Blessing, C., Mandemaker, I.K., Gonzalez-Leal, C., Preisser, J., Schomburg, A. and Ladurner, A.G. (2020) The oncogenic helicase ALC1 regulates PARP inhibitor potency by trapping PARP2 at DNA breaks. Mol. Cell 80, 862–75.e6 10.1016/j.molcel.2020.10.009 33275888

[166] Gomez, M.K., Illuzzi, G., Colomer, C., Churchman, M., Hollis, R.L., O'Connor, M.J. et al. (2020) Identifying and overcoming mechanisms of PARP inhibitor resistance in homologous recombination repair-deficient and repair-proficient high grade serous ovarian cancer cells. Cancers (Basel) 12, 1503 10.3390/cancers12061503 32526907 PMC7353027

[167] Drown, B.S., Shirai, T., Rack, J.G.M., Ahel, I. and Hergenrother, P.J. (2018) Monitoring poly(ADP-ribosyl)glycohydrolase activity with a continuous fluorescent substrate. Cell Chem. Biol. 25, 1562–70.e19 10.1016/j.chembiol.2018.09.008 30318463 PMC6309520

[168] Liu, X., Xie, R., Yu, L.L., Chen, S.H., Yang, X., Singh, A.K. et al. (2020) AI26 inhibits the ADP-ribosylhydrolase ARH3 and suppresses DNA damage repair. J. Biol. Chem. 295, 13838–13849 10.1074/jbc.RA120.012801 32753484 PMC7535916

[169] Waszkowycz, B., Smith, K.M., McGonagle, A.E., Jordan, A.M., Acton, B., Fairweather, E.E. et al. (2018) Cell-active small molecule inhibitors of the DNA-damage repair enzyme poly(ADP-ribose) glycohydrolase (PARG): discovery and optimization of orally bioavailable quinazolinedione sulfonamides. J. Med. Chem. 61, 10767–10792 10.1021/acs.jmedchem.8b01407 30403352

[170] Pillay, N., Tighe, A., Nelson, L., Littler, S., Coulson-Gilmer, C., Bah, N. et al. (2019) DNA replication vulnerabilities render ovarian cancer cells sensitive to poly(ADP-Ribose) glycohydrolase inhibitors. Cancer Cell 35, 519–33.e8 10.1016/j.ccell.2019.02.004 30889383 PMC6428690

[171] Chen, S.H. and Yu, X. (2019) Targeting dePARylation selectively suppresses DNA repair-defective and PARP inhibitor-resistant malignancies. Sci. Adv. 5, eaav4340 10.1126/sciadv.aav4340 30989114 PMC6457938

[172] Houl, J.H., Ye, Z., Brosey, C.A., Balapiti-Modarage, L.P.F., Namjoshi, S., Bacolla, A. et al. (2019) Selective small molecule PARG inhibitor causes replication fork stalling and cancer cell death. Nat. Commun. 10, 5654 10.1038/s41467-019-13508-4 31827085 PMC6906431

[173] Yu, M., Chen, Z., Zhou, Q., Zhang, B., Huang, J., Jin, L. et al. (2022) PARG inhibition limits HCC progression and potentiates the efficacy of immune checkpoint therapy. J. Hepatol. 77, 140–151 10.1016/j.jhep.2022.01.026 35157958

[174] Coulson-Gilmer, C., Morgan, R.D., Nelson, L., Barnes, B.M., Tighe, A., Wardenaar, R. et al. (2021) Replication catastrophe is responsible for intrinsic PAR glycohydrolase inhibitor-sensitivity in patient-derived ovarian cancer models. J. Exp. Clin. Cancer Res. 40, 323 10.1186/s13046-021-02124-0 34656146 PMC8520217

[175] Gravells, P., Grant, E., Smith, K.M., James, D.I. and Bryant, H.E. (2017) Specific killing of DNA damage-response deficient cells with inhibitors of poly(ADP-ribose) glycohydrolase. DNA Repair (Amst) 52, 81–91 10.1016/j.dnarep.2017.02.010 28254358 PMC5360195

[176] Schuller, M. and Ahel, I. (2022) Beyond protein modification: the rise of non-canonical ADP-ribosylation. Biochem. J. 479, 463–477 10.1042/BCJ20210280 35175282 PMC8883491

[177] Nagy, Z., Kalousi, A., Furst, A., Koch, M., Fischer, B. and Soutoglou, E. (2016) Tankyrases promote homologous recombination and check point activation in response to DSBs. PLoS Genet. 12, e1005791 10.1371/journal.pgen.1005791 26845027 PMC4741384

[178] Dhoonmoon, A., Nicolae, C.M. and Moldovan, G.L. (2022) The KU-PARP14 axis differentially regulates DNA resection at stalled replication forks by MRE11 and EXO1. Nat. Commun. 13, 5063 10.1038/s41467-022-32756-5 36030235 PMC9420157

[179] Gogola, E., Duarte, A.A., de Ruiter, J.R. et al. (2019) Selective Loss of PARG Restores PARylation and Counteracts PARP Inhibitor-Mediated Synthetic Lethality. Cancer Cell 35, 950–952 10.1016/j.ccell.2019.05.012 31185216 PMC6561720

[180] Demin, A.A., Hirota, K., Tsuda, M. et al. (2021) XRCC1 prevents toxic PARP1 trapping during DNA base excision repair. Mol. Cell 81, 3018–3030.e5 10.1016/j.molcel.2021.05.009 34102106 PMC8294329

